# The effects of non-andrological medications on erectile dysfunction: a large single-center retrospective study

**DOI:** 10.1007/s40618-023-02011-9

**Published:** 2023-01-19

**Authors:** R. Mazzilli, V. Zamponi, F. Mangini, S. Olana, G. Defeudis, A. Faggiano, D. Gianfrilli

**Affiliations:** 1 Endocrinology and Andrology Unit, Department of Clinical and Molecular Medicine, Sapienza University of Rome, Sant’Andrea Hospital, Via di Grottarossa 1036-1039, 00100 Rome, Italy; 2 Unit of Endocrinology and Diabetes, Department of Medicine, University Campus Bio-Medico di Roma, Rome, Italy; 3 Endocrinology and Andrology Unit, Department of Experimental Medicine, Sapienza University of Rome, Rome, Italy

**Keywords:** Medications, Drugs, Erectile dysfunction, Andrological diseases, Diabetes mellitus

## Abstract

**Purpose:**

To evaluate the association among andrological diseases at the first outpatient visit and the medications taken by patients for other comorbidities, as well as the differential impact between specific medication and relative comorbidities.

**Methods:**

This is a single-center retrospective study based on subjects who referred to the Andrology Unit with a well-defined andrological diagnosis.

**Results:**

A total of 3752 subjects were studied (mean age ± DS 46.2 ± 16.5 years). A total of 19 categories of andrological diseases and 110 type of medications for other comorbidities were identified. ED was the most frequent andrological pathology at the first andrological examination (28.7%), followed by infertility (12.4%). The couple of variables that were statistically significant in the univariate association analysis (*p* < 0.001) were: ED and (a) antihypertensives; (b) antihyperglycemics; (c) lipids-lowering; (d) psychotropics. The univariate and multivariate regression analyses confirmed the association. All the related comorbidities were also significantly associated with the univariate analysis, and all remained significantly associated with multivariate analysis. A multivariate analysis was also conducted to analyze the association between ED and the following pairs of variables “DM-antihyperglycemics”, “dyslipidemia-lipids-lowering”, and “hypertension-antihypertensives”. In all cases, the pathology, but not the specific treatment, was significantly associated with ED.

**Conclusion:**

ED is significantly associated with antihypertensive, antihyperglycemic, lipid-lowering, psychotropic drugs’ intake. Anyway, ED appears to be more related to the diseases than to the specific therapies. The definitive cause/effect relationship should be established based on future prospective studies.

## Introduction

The disorders that lead patients to carry out an andrological evaluation can be various and heterogeneous, i.e., sexual and ejaculatory dysfunction, infertility, hypogonadism, and infections; frequently, an etiopathogenetic cause cannot be identified. Several studies have hypothesized a correlation between certain medications and andrological diseases [1–3].

Considering sexual dysfunction, the classes of drugs most associated with these disorders are psychotropics and antihypertensives [1, 4]. Considering the formers, antipsychotics are associated with ED [5], reduced orgasm intensity and ejaculatory disorders; this could be explained by inhibitory action on dopaminergic receptors resulting in hyperprolactinemia and hypogonadism, and by the antihistamine, anticholinergic, and alpha-adrenergic action that could reduce peripheral vasodilation. Furthermore, antidepressants can induce decrease of libido, delay in reaching orgasm, and less frequently, reduced arousal. Pharmacological treatment of hypertension may adversely affect sexual function and accumulating evidence supports the detrimental role of beta-blockers on erectile function [4].

Anyway, anti-androgens, luteinizing hormone-releasing agonists, and antagonists used to treat prostate cancer, anti-ulcer drugs, opiates, and digoxin have also been linked with ED [1].

Considering male fertility, several medications have been shown to have adverse effects, acting through different mechanisms, including direct effects on germ cells, effects on the hypothalamus pituitary gonadal axis as well as effects on erectile or ejaculatory function [3]. In fact, in the management of infertile couples, the potential negative impact on seminal parameters of any drugs taken by patients should be considered [6]. In this regard, chemo- and radio-therapy certainly affect semen parameters; the damage to spermatogenesis is dependent on the dose received and the age of the patient [7, 8].

In addition, several studies have shown that the intake of psychotropic drugs can determine alteration of semen quality, mainly a significant impairment of progressive motility and sperm morphology [9]. Finally, androgens, anti-androgens, and opioids have also been linked with male infertility [8].

The purpose of this study was to evaluate the association among the andrological diseases at the first outpatient visit and the medications taken by patients for other comorbidities, as well as the differential impact between specific medications and relative comorbidities.

## Materials and methods

### Population

In this single-center retrospective study, male subjects, Caucasian, aged between 18 and 45 years, who referred to the Andrology Unit of Sant’Andrea University Hospital from January 2013 to December 2020 were evaluated. All patients with an andrological diagnosis were considered eligible for statistical analysis, using a specific electronic database adapted to the Andrology Unit. The prevalence of first diagnosed andrological pathology and ongoing chronic medications for non-andrological comorbidities were assessed.

### Statistical analysis

Categorical variables were expressed as an absolute number and percentage. Continuous variables as mean ± standard deviation (SD). The Chi-squared test was performed to identify associations between different variables. A *p* value < 0.05 was considered statistically significant.

A logistic regression model was adopted to evaluate the impact of classes of medications on andrological diseases (Table 1), considering Odds ratio (OR), *p* value, and 95% confidence interval [95% CI].

**Table 1:** Andrological diseases and macro-categories of medications evaluated

Andrological diseases		
Erectile dysfunction	Genetic disorders	Decreased libido
Infertility	Penile phimosis	Hypogonadism
Ejaculatory and sexual disorders (delayed ejaculation/retrograde ejaculation/premature ejaculation/painful ejaculation, anejaculation and anorgasmia)	Hydrocele	Infectious diseases (HIV, HPV, balanoposthitis)
Inflammatory diseases (orchitis, epididymitis, testicular pain, and epididymal and testicular cysts)	Haemospermia	Dysmorphia e gender identity disorders
Varicocele	Gynecomastia	Benign prostatic hypertrophy
Induratio penis plastica	Cryptorchidism	
Hyperprolactinemia	Testicular tumors	
Macro-categories of medications		
Addictive	Antiulcer	Immunological therapies
Adrenergic of the respiratory system	Antivertiginous	Immunosuppressants
Adrenergic of the respiratory system and corticosteroids	Antiviral	Leukemia therapy
Alpha lytic	Antiarrhythmics	Lipid-lowering
Antiacids	Bile acid sequestrants	Male fertility supplements
Anti migraineurs	Biphosphonates	Medications for pain therapy
Anti gouty	Capillaroprotectors	Mesalamine
Antianginal	Cardioaspirin	Muscle relaxants
Antiasthmatics	Corticosteroids	N-acetyl-cysteine
Antibiotics	Decongestants	Nitrates
Anticholinergic	Dermatological drugs	Oxygen therapy
Anticoagulant	Digital	Pancreatic enzymes
Antiemetics	Diuretics	Paracetamol
Antifungals	Drugs for dementia	Parkinson’s drugs
Antihemorrhagic	Electrolytes	Peripheral vasodilators
Antihistamines	Erythropoietin	Phosphodiesterase 5 inhibitors
Antihyperglycemic	FANS	Prokinetics
Antihypertensive	Ferrochelanting drugs	Psychotropic drugs
Antileukotrienes	Folic acid	Sulfasalazine
Antimalarial	Gastroprotectors	Urinary antispasmodics
Antineoplastic antimetabolites	Glaucoma’s therapy	Urinary lithogenic
Antiplatelet	HIV/HBV/HCV therapy	Ursodeoxycholic acid
Antispasmodics	Hormonal therapy	Vitamins B, B12, C, D, E

A univariate analysis and a multivariate analysis were also conducted to evaluate the effect of medications and the related comorbidities on andrological disease which resulted significantly associated.

Statistical analysis was performed using the IBM-SPSS 25 version (IBM Corporation, New York, United States of America).

### Ethics

The study adhered to the Hospital’s Ethics Committee guidelines and to the Ethical Principles for Medical Research Involving Human Subjects as adopted at the 18th WMA General Assembly, Helsinki, Finland, June 1964, and amended by the 55th WMA General Assembly, Tokyo, Japan, October 2004 and subsequent modifications when enforced (last, Fortaleza, Brazil, October 2013).The study was approved by the Ethics Committee of the Hospital (Protocol n. RIF. CE 6559_2021).

## Results

The study population included 3752 subjects; the mean age ± DS was 46.2 ± 16.5 years. The occupational status was available for 1427 patients, of which 34.9% were self-employed/freelancers, 32.9% employees, 13.6% retirees, 10.1% unemployed, and 8.5% students. The marital status was available for 2464 patients, of which 57.1% were married/cohabitant, 29.5% were in a relationship, and 13.3% were single.

**Fig. 1:**
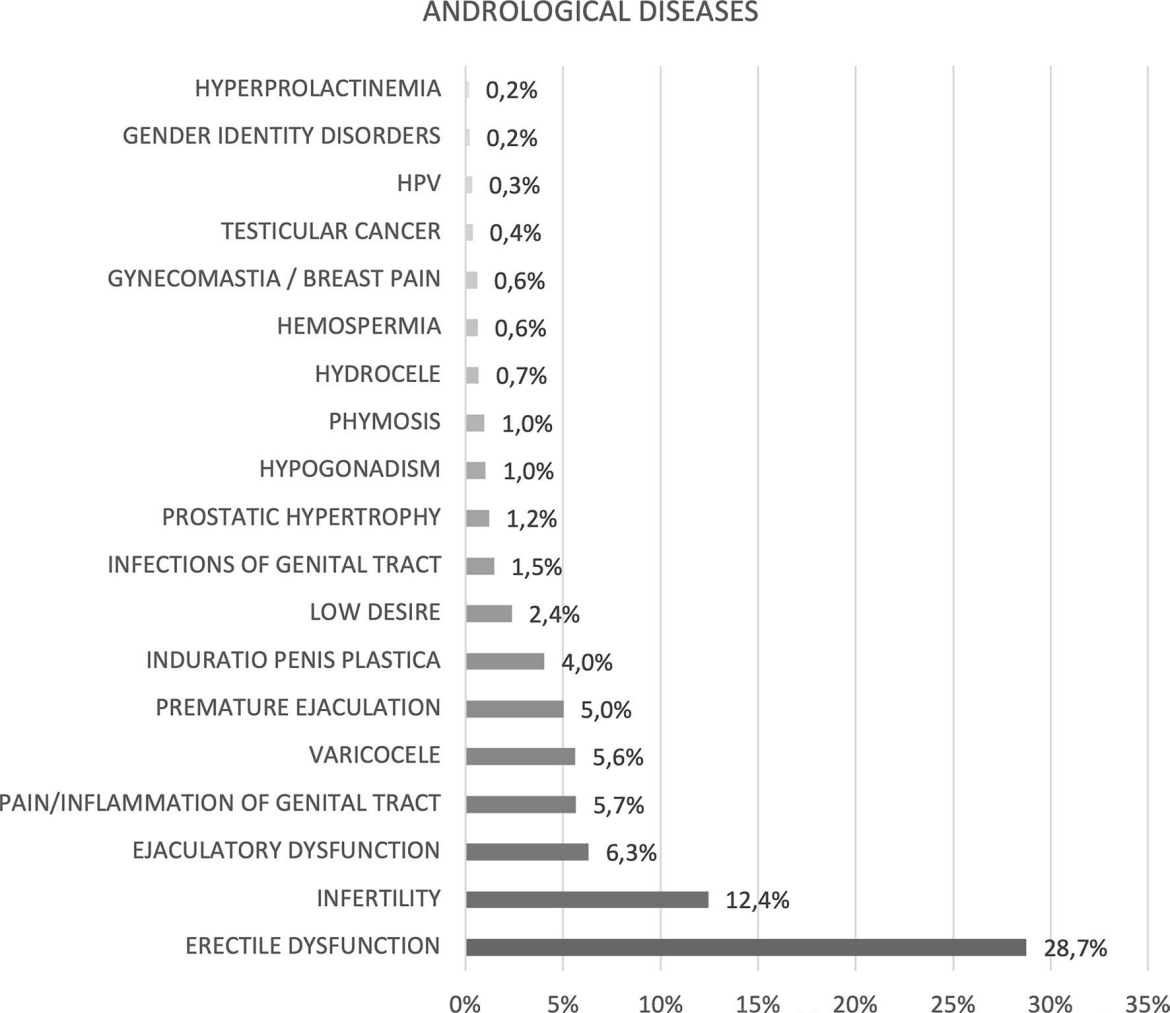
Prevalence of the main andrological diseases

**Fig. 2:**
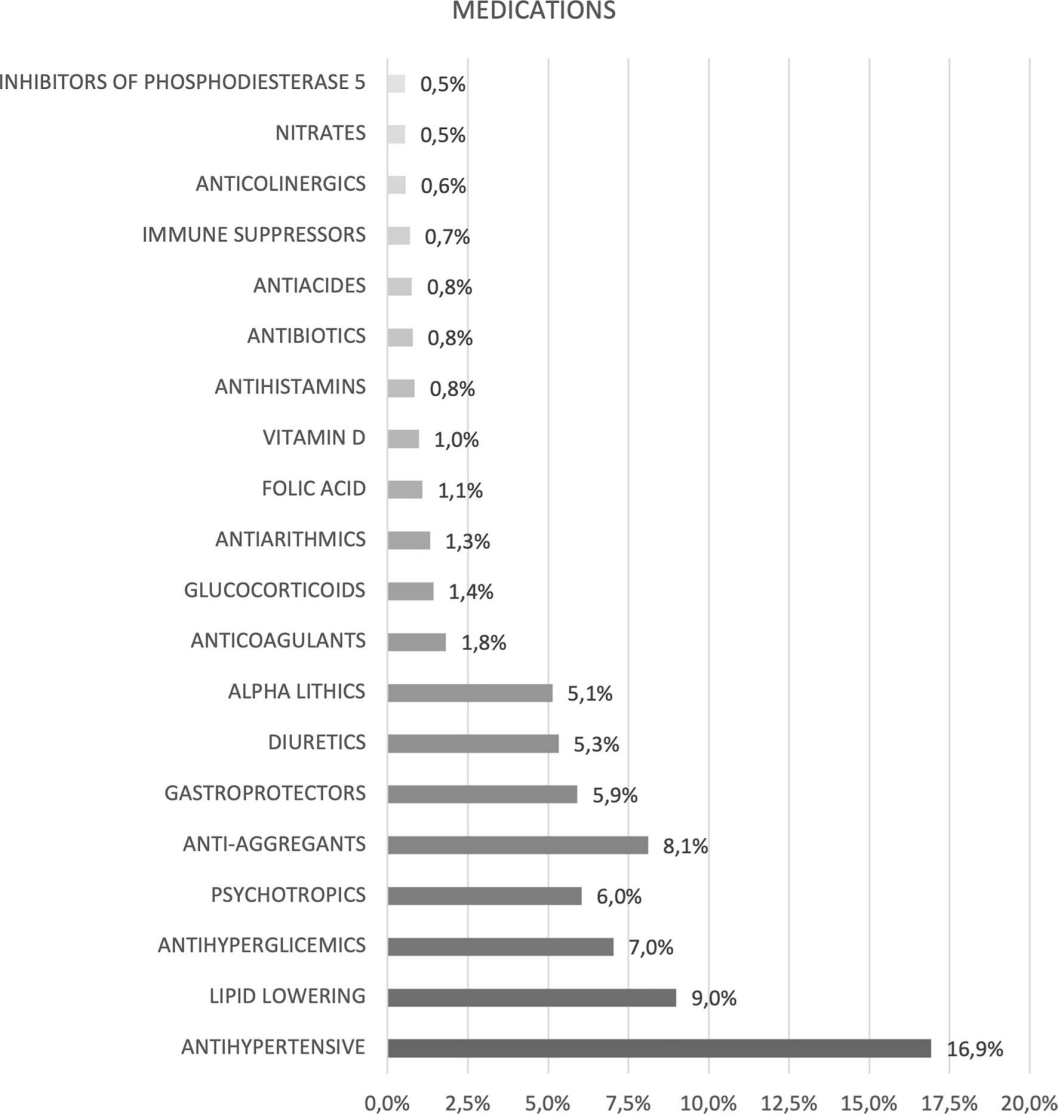
Prevalence of the main medications for comorbidities

A total of 19 categories of andrological pathologies (Fig. 1) and 110 type of medications for comorbidities (*n* 110 drugs grouped into *n* 69 categories) (Fig. 2) were identified.

ED was the most frequent andrological pathology at the first andrological examination (28.7%), followed by infertility (12.4%). The mean age ± SD of ED patients was significantly higher than the remaining population (54.3 ± 13.7 vs 42.9 ± 16.4 years, *p* < 0.001).

The most frequent macro-category of medications found in the population was antihypertensive (16.9%), followed by lipid-lowering (9.0%) and antihyperglycemic (7.0%).

The couple of variables that were statistically significant in the univariate association analysis (*p* < 0.001) were: (a) antihypertensive and ED; (b) antihyperglycemic and ED; (c) lipid-lowering and ED; (d) psychotropics and ED.

### Macro-categories of medications and ED

Specifically, antihyperglycemic (*n* 294 patients; OR 3.7, 95% CI 2.90–4.83, *p* < 0.001), lipid-lowering (*n* 354 patients; OR 2.94, 95% CI 2.34–3.69, *p* < 0.001), antihypertensive (*n* 707 patients; OR 3.9, 95% CI 3.32–4.71, *p* < 0.001), and psychotropic (*n* 248 patients; OR 2.3, 95% CI 1.73–2.93, *p* < 0.001). All these drug categories remained significantly associated with the multivariate analysis: antihyperglycemic (OR 2.1, 95% CI 1.59–2.80, *p* < 0.001), lipid-lowering (OR 1.3, 95% CI 1.00–1.70, *p* = 0.04), antihypertensive (OR 3.1, 95% CI 2.54–3.75, *p* < 0.001), and psychotropic drugs (OR 2.0, 95% CI 1.48–2.57, *p* < 0.001). None of the other variables resulted significantly associated.

### Specific drugs among macro-categories of medications and ED

Considering the specific drugs, among macro-categories of medications, a significant correlation has been found between ED and specific drugs among medications’ macro-categories, such as: (i) metformin, insulin, glinides, DPP4 inhibitors, and sulfonylureas among antihyperglycemic; (ii) statins among lipid-lowering; (iii) beta-blockers, calcium channel antagonists, ACE inhibitors, angiotensin receptor antagonists (sartans) among antihypertensive, and (iv) benzodiazepines and SSRI among psychotropic (*p* < 0.5) (Table 2).

**Table 2:** Antihypertensive, antihyperglycemic drugs and erectile dysfunction

Medications	Coeff	*p* value Correlation test	*p* value Chi-squared test
Metformin	0.15	< 0.001	< 0.001
Insulin	0.12	< 0.001	< 0.001
Glinides	0.08	< 0.001	< 0.001
DPP4 inhibitors	0.07	< 0.001	< 0.001
Sulfonylureas	0.05	< 0.001	< 0.001
Statins	0.21	< 0.001	< 0.001
Beta-blockers	0.17	< 0.001	< 0.001
Calcium channel antagonists	0.15	< 0.001	< 0.001
ACE inhibitors	0.16	< 0.001	< 0.001
Angiotensin receptor antagonists	0.19	< 0.001	< 0.001
Benzodiazepines	0.21	0.002	0.002
SSRI	0.26	< 0.001	< 0.001

*DPP4* dipeptidyl peptidase-4, *ACE* angiotensin-converting enzyme, *SSRI* selective serotonin reuptake inhibitors

### Related comorbidities of medications and ED

All the related comorbidities were also significantly associated with the univariate analysis: DM (*n* 332 patients; OR 3.5, 95% CI 2.76–4.45, *p* < 0.001), dyslipidemia (*n* 395 patients; OR 3.0, 95% CI 2.39–3.70, *p* < 0.001), hypertension (*n* 762 patients; OR 4.0, 95% CI 3.39–4.77, *p* < 0.001), psychiatric disorders (*n* 248 patients; OR 2.3, 95% CI 1.73–2.93, *p* < 0.001), and all remained significantly associated with multivariate analysis: DM (OR 1.9, 95% CI 1.47–2.49, *p* < 0.001), dyslipidemia (OR 1.4, 95% CI 1.06–1.76, *p* = 0.02), hypertension (OR 3.1, 95% CI 2.58–3.77, *p* < 0.001), and psychiatric disorders (OR 2.0, 95% CI 1.48–2.57, *p* < 0.001).

A multivariate analysis was also conducted to analyze the association between ED and the following pairs of variables “DM-antihyperglycemics”, “dyslipidemia-lipids-lowering”, and “hypertension-antihypertensives”. In all cases, the pathology, but not the specific treatment, was significantly associated to ED: DM (OR 2.0, 95% CI 1.07–3.86, *p* = 0.03)—antihyperglycemic (OR 1.86, 95% CI 0.94–3.67, *p* = 0.08); dyslipidemia (OR 2.9, 95% CI 1.53–5.41, *p* = 0.001)—lipid-lowering (OR 1.04, 95% CI 0.53–2.01, *p* = 0.9); hypertension (OR 3.4, 95% CI 1.97–5.88, *p* < 0.001)—antihypertensives (OR 1.2, 95% CI 1.68–2.10, *p* = 0.5).

The analysis “psychiatric-psychotropic disorder” was not possible due to the absence of patients with psychiatric disorders without therapy.

**Fig. 3:**
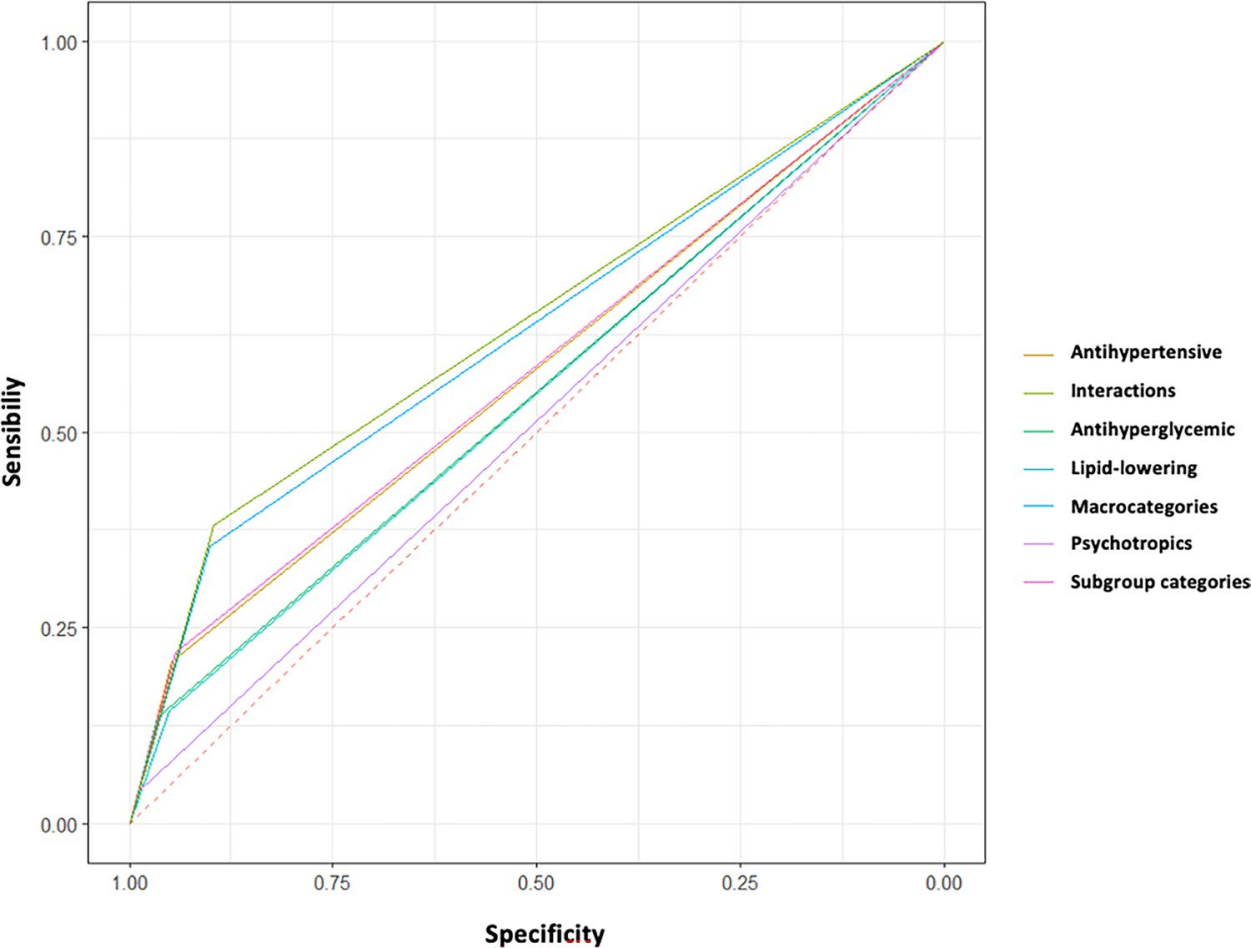
ROC curves of the logistic regression models for erectile dysfunction

Finally, the ROC curves of the logistic regression models for ED were analyzed; the model that considered interactions between drug macro-categories was the predominant (Fig. 3).

## Discussion

In this study, the possible association between the andrological diseases of patients who referred to the Andrology Unit and the medications taken by the patients for other comorbidities was investigated.

ED was found to be the most frequent andrological disease. The age of ED patients was significantly higher than the other population, according to previous studies [10]. In this regard, McMahon et al. estimated that the prevalence of ED was 5% among 40-year-old men, 10% among 60-year-olds, and 15% between 70 and 30–40% among 80-year-olds [10].

Considering the association between andrological pathologies and medications, the statistical analysis was focused on ED, due to the heterogeneity, the reduced sample, and the scarce significance of the results for the association with the other diseases.

In our study, ED was associated with different categories of medications, chronically used by patients at the time of the first andrological evaluation, and the associations that we found are consistent with other studies in the literature. In fact, some drugs among the macro-categories of antihypertensive, antihyperglycemic, lipid-lowering [11], and psychotropic drugs [12, 13] are associated with the onset of ED. Analyzing the different subcategories of drugs, we found that the use of beta-blockers, calcium channel antagonists, diuretics, ace inhibitors, sartans, antidepressants (SSRIs), benzodiazepines, insulin, repaglinide, metformin, statins, and fibrates are associated with ED.

Considering antihypertensives, Hernandez-Cerda J et al. found in their observational cross-sectional study a prevalence of ED in 71% of male patients with hypertension treated for at least 6 months with beta-blockers [14].

Conversely, the correlation between ED and antihyperglycemic drugs was less investigated, since most of the trials were carried out on animals and not on humans [15, 16]. Giagulli et al. [17] conducted a retrospective observational study on 43 obese men with ED, DM, and hypogonadism. Patients were treated with testosterone undecanoate (1000 mg every 12 weeks) and metformin (2–3 mg per day) for 1 year; in patients whose glycemic target was not reached, a Glucagon like peptide-1 antagonist (GLP-1 a) was added for further 12 months. The authors observed that patients treated with liraglutide showed a significant improvement in erectile function compared to the group that received only testosterone and metformin; therefore, this finding underlines a potential protective effect of GLP-1a on sexual function. Similar results were observed by Defeudis et al. [18], who found in a prospective observational study that patients treated with GLP-1a showed a better erectile function than patients treated with insulin; the results were also confirmed after adjustment for age and duration of DM. Nevertheless, diabetes is closely related to cardiovascular disease [19].

Considering lipids-lowering, mainly statins, data currently available from randomized trials seem to indicate a small improvement in erectile function [11].

On the other hand, statins can worsen erectile function blocking HMG-CoA, and they inhibit the production of cholesterol, which is the substrate for the formation of testosterone; however, further studies are needed to clarify this result. Nevertheless, regarding psychotropic drugs, a recent study conducted by Trincheri et al. highlighted that antipsychotic and antidepressant are associated with ED [13]. Among antipsychotics, first-generation antipsychotics like risperidone or paliperidone severely affect sexual function, due to dopamine antagonism (which can result in hyperprolactinemia), histaminergic, cholinergic, and alpha-adrenergic effects, as well as serotonin-mediated sexual demotivation [20]. In this regard, sexual function should be measured and monitored in patients treated with psychotropics and primary prevention should be conducted when choosing drugs, to preserve sexuality [20].

However, some of the medications that we found to be associated with ED have not been evaluated, and could be considered new evidence or, more likely, the consequence of the underlying comorbidity more than specific therapy. In fact, the pathogenetic mechanisms underlying ED are the same as those of hypertension, DM, dyslipidemia, and psychiatric diseases [21]. In particular, regarding the latter aspect, the multidimensional nature of ED should always be taken into consideration, which can be caused by organic and/or psychological [12]. Furthermore, the recent global outbreak of coronavirus disease (COVID-19) caused by the severe acute respiratory syndrome coronavirus 2 (SARS-CoV-2) contributes to increase in the last years the development of psychiatric disorders as well as sexual and reproductive health issues, mainly ED [22]. In fact, ED and COVID-19 share similar risk factors and recent evidence highlighted that in subjects affected by COVID-19, the risk of ED occurrence is 3.3 times higher, even after adjustment for known risk factors [23]. An andrological evaluation and a tailored treatment should be considered during the follow-up of COVID-19 survivors, including an adequate psychological support systems and a promotion of sexual health, both in patients and in healthcare workers operating in COVID-19 environments [22, 24].

In light of all these considerations, the associations between ED and the medications investigated should be partly due to the underlying pathology.

To evaluate and confirm this aspect, the statistically significant association between the drug categories (antihyperglycemic, antihypertensive, and lipid-lowering) and ED is accompanied by a similar statistically significant association between ED and underlying comorbidities (DM, hypertension, and dyslipidemia). Furthermore, the multivariate analysis of the association between ED and the pairs of variables consisting of a specific comorbidity and the respective drug therapy (i.e., DM and antihyperglycemic or hypertension and antihypertensive drugs), highlighted statistical significance of the association between ED and comorbidity, but not with therapy. Nevertheless, the analysis could be affected by a collinearity bias given the relationship between each comorbidity and the related drug treatment.

Finally, regarding the model that considers the interactions between different drugs, interesting results were found for what concern the interaction between antihypertensives, lipids-lowering, and antihyperglycemic drugs. This could be due to that the simultaneous consumption of drugs, and consequently of the underlying pathologies, increases the risk of developing the ED itself.

The main limitations of the study are the retrospective nature that cannot allow to evaluate the specific molecules of individual drug class categories for all patients, and the difficulty to identify the direct causality between drug intake and ED, since ED shares the same pathogenetic mechanisms as DM, dyslipidemia, arterial hypertension, as well as psychiatric pathologies.

## Conclusions

In conclusion, ED appears to be the most prevalent pathology in the andrological population. Furthermore, ED was significantly associated with antihypertensive, antihyperglycemic, lipid-lowering, psychotropic drugs’ intake; the simultaneous use of several categories of these drugs was associated with an increased risk of developing ED itself. Anyway, our results highlighted that ED is more related to the diseases than to the specific therapies. The definitive cause-and-effect relationship between these medications and ED can be established based on future prospective studies.

## Data Availability

If requested.

## References

[1] Francis ME, Kusek JW, Nyberg LM, Eggers PW (2007). The contribution of common medical conditions and drug exposures to erectile dysfunction in adult males. J Urol.

[2] Yafi FA, Jenkins L, Albersen M, Corona G, Isidori AM, Goldfarb S, Maggi M, Nelson CJ, Parish S, Salonia A, Tan R, Mulhall JP, Hellstrom WJ (2016). Erectile dysfunction. Nat Rev Dis Primers.

[3] Nudell DM, Monoski MM, Lipshultz LI (2002). Common medications and drugs: how they affect male fertility. Urol Clin North Am.

[4] Manolis A, Doumas M, Ferri C, Mancia G (2020). Erectile dysfunction and adherence to antihypertensive therapy: focus on β-blockers. Eur J Intern Med.

[5] Isidori AM, Giammusso B, Corona G, Verze P (2019). Diagnostic and therapeutic workup of erectile dysfunction: results from a delphi consensus of andrology experts. Sex Med.

[6] Elia J, Imbrogno N, Delfino M, Mazzilli R, Spinosa V, Mazzilli F (2013). Impact of long-term and short-term therapies on seminal parameters. Arch Ital Urol Androl.

[7] Pallotti F, Pelloni M, Faja F, Di Chiano S, Di Rocco A, Lenzi A, Lombardo F, Paoli D (2021). Semen quality in non-Hodgkin lymphoma survivors: a monocentric retrospective study. Hum Reprod.

[8] Velez D, Ohlander S (2021). Medical therapies causing iatrogenic male infertility. Fertil Steril.

[9] Mazzilli R, Curto M, De Bernardini D, Olana S, Capi M, Salerno G, Cipolla F, Zamponi V, Santi D, Mazzilli F, Simmaco M, Lionetto L (2021). Psychotropic drugs levels in seminal fluid: a new therapeutic drug monitoring analysis?. Front Endocrinol (Lausanne).

[10] McMahon CG (2019). Current diagnosis and management of erectile dysfunction. Med J Aust.

[11] Kostis JB, Dobrzynski JM (2019). Statins and erectile dysfunction. World J Mens Health.

[12] Mazzilli R, Angeletti G, Olana S, Delfino M, Zamponi V, Rapinesi C, Del Casale A, Kotzalidis GD, Elia J, Callovini G, Girardi P, Mazzilli F (2018). Erectile dysfunction in patients taking psychotropic drugs and treated with phosphodiesterase-5 inhibitors. Arch Ital Urol Androl.

[13] Trinchieri M, Trinchieri M, Perletti G, Magri V, Stamatiou K, Cai T, Montanari E, Trinchieri A (2021). Erectile and ejaculatory dysfunction associated with use of psychotropic drugs: a systematic review. J Sex Med.

[14] Hernández-Cerda J, Bertomeu-González V, Zuazola P, Cordero A (2020). Understanding erectile dysfunction in hypertensive patients: the need for good patient management. Vasc Health Risk Manag.

[15] Corona G, Isidori AM, Aversa A, Bonomi M, Ferlin A, Foresta C, La Vignera S, Maggi M, Pivonello R, Vignozzi L, Lombardo F (2020). Male and female sexual dysfunction in diabetic subjects: focus on new antihyperglycemic drugs. Rev Endocr Metab Disord.

[16] Defeudis G, Mazzilli R, Di Tommaso AM, Zamponi V, Carlomagno F, Tuccinardi D, Watanabe M, Faggiano A, Gianfrilli D (2022). Effects of diet and antihyperglycemic drugs on erectile dysfunction: a systematic review. Andrology.

[17] Giagulli VA, Carbone MD, Ramunni MI, Licchelli B, De Pergola G, Sabbà C, Guastamacchia E, Triggiani V (2015). Adding liraglutide to lifestyle changes, metformin and testosterone therapy boosts erectile function in diabetic obese men with overt hypogonadism. Andrology.

[18] Defeudis G, Di Tommaso AM, Di Rosa C, Cimadomo D, Khazrai YM, Faggiano A, Cincione RI, Napoli N, Mazzilli R (2022). The role of antihyperglycemic drugs and diet on erectile function: results from a perspective study on a population with prediabetes and diabetes. J Clin Med.

[19] Pofi R, Giannetta E, Feola T, Galea N, Barbagallo F, Campolo F, Badagliacca R, Barbano B, Ciolina F, Defeudis G, Filardi T, Sesti F, Minnetti M, Vizza CD, Pasqualetti P, Caboni P, Carbone I, Francone M, Catalano C, Pozzilli P, Lenzi A, Venneri MA, Gianfrilli D, Isidori AM (2022). Sex-specific effects of daily tadalafil on diabetic heart kinetics in RECOGITO, a randomized, double-blind, placebo-controlled trial. Sci Transl Med..

[20] Jannini TB, Sansone A, Rossi R, Di Lorenzo G, Toscano M, Siracusano A, Jannini EA (2022). Pharmacological strategies for sexual recovery in men undergoing antipsychotic treatment. Expert Opin Pharmacother.

[21] Mobley DF, Khera M, Baum N (2017). Recent advances in the treatment of erectile dysfunction. Postgrad Med J.

[22] Sansone A, Mollaioli D, Ciocca G, Limoncin E, Colonnello E, Vena W, Jannini EA (2021). Addressing male sexual and reproductive health in the wake of COVID-19 outbreak. J Endocrinol Invest.

[23] Katz J, Yue S, Xue W, Xue W, Gao H (2022). Increased odds ratio for erectile dysfunction in COVID-19 patients. J Endocrinol Invest.

[24] Pizzol D, Shin JI, Trott M, Ilie PC, Ippoliti S, Carrie AM, Ghayda RA, Lozano JMO, Muyor JM, Butler L, McDermott DT, Barnett Y, Markovic L, Grabovac I, Koyanagi A, Soysal P, Tully MA, Veronese N, Smith L (2022). Social environmental impact of COVID-19 and erectile dysfunction: an explorative review. J Endocrinol Invest.

